# Supplementary material for: Viral susceptibility across host species is largely independent of dietary protein to carbohydrate ratios
Source: J Evol Biol. 2021 Mar 31;34(5):746–56. doi: 10.1111/jeb.13773 (PMC8436156; doi:10.1111/jeb.13773)
Supplement: Supplementary file 1 — Supplementary Material [file JEB-34-746-s002.pdf]

Diet has no effect on susceptibility to an RNA virus across host species

Supplementary Table: List of species used in the experiment and their rearing food for stock populations. All cornmeal and Proprionic medium have dried yeast sprinkled onto the surface of the food, other food types do not unless stated below. Stock lines are the Drosophila Species Stock Centre strain IDs, Cambridge refers to flies from stocks at the Genetics Department University of Cambridge. Flies are from the following genera: Drosophila, Scaptodrosophila , Hirtodrosophila or Zaprionous.

Species	Rearing food	Stock line
D.americana	Malt	15010-0951.20
D.ananassae	Cornmeal	Cambridge
D.arizonae	Banana	15081-1271.26
D.buzzatii	Malt	15081-1291.01
D.euronotus	Cornmeal	15030-1131.01
D.flavomontana	Malt + yeast	15010-0981.05
D.hydei	Cornmeal	15085-1641.71
D.lacicola	Malt	15010-0991.17
D.mauritiana	Proprionic	Cambridge
D.melanogaster	Cornmeal	Cambridge
D.micromelanica	Cornmeal	15030-1151.01
D.montana	Malt + yeast	Cambridge
D.prosaltans	Proprionic	14045-0901.02
D.saltans	Cornmeal	Cambridge
D.santomea	Cornmeal	14021-0271.00
D.simulans	Cornmeal	Cambridge
D.subobscura	Cornmeal	14011-0131.15
D.virilis	Proprionic	Cambridge
H.duncani	Proprionic	92000-0075.00
S. lativittata	Banana	11020-0081.00
S.lebanonensis	Proprionic	Cambridge
S.pattersoni	Banana	11010-0031.00

Z. davidi	Banana	50001-1040.00
Z. taronus	Banana	50001-1020.00
Z. tuberculatus	Banana	50001-0001.05
D. putrida	Cornmeal	15150-2101.00
D. tropicalis	Cornmeal	14030-0801.01

Food recipes can be found in:

1. **Longdon B, Hadfield JD, Day JP, Smith SC, McGonigle JE, Cogni R, et al. The Causes and Consequences of Changes in Virulence following Pathogen Host Shifts. PLoS Pathog. 2015;11(3):e1004728. Epub 2015/03/17. doi: 10.1371/journal.ppat.1004728. PubMed PMID:25774803.**
2. **Longdon B, Hadfield JD, Webster CL, Obbard DJ, Jiggins FM. Host Phylogeny Determines Viral Persistence and Replication in Novel Hosts. Schneider DS, editor. PLoS Pathog. (2011) ;7: e1002260. doi:10.1371/journal.ppat.1002260**

Supplementary Table: Food recipes and nutrient breakdown

Laboratory Normal Cornmeal Diet					
	Cornmeal	Dextrose (g)	Yeast	Agar	Totals
	88	88	19	11	206
Kcal	325.6	321.2	67.45	2.86	717.11
Fat (g)	2.464	0.088	1.52	0	4.072
Carbohydrate (g)	69.52	83.6	3.61	0.77	157.5
Protein (g)	6.16	0	8.265	0.055	14.48
Salt (g)	0	0	0.024795	0	0.024795
Sugars (g)	1.408	83.6	2.66	0.033	87.701

Diet	Ratio Protein: Carb	Cornmeal (g)	Dextrose (g)	Yeast (g)	Agar (g)	Nipagin (ml)	dH20 (L)	Calories per 100ml
High	1:06	176	131.2	84	22	29	1	142.60
Medium	1:10	176	176	38	22	29	1	142.30
Low	1:20	176	203	10	22	29	1	142.02

Supplementary Table: q-PCR RpL32 primer sequences for the fly species used in the experiment

Primer Type	Primer Name	Sequence
**Forward Primers**	F-a	TGCCAAGTTGTCGCACAAATGG
	F-b	TGCTAAGTTGTCGCACAAATGG
	F-c	TGCCAAGCTGTCGCACAAATGG
	F-d	TGCTAAGCTGTCGCACAAATGG
	F-e	TGCGAAGTTGTCGCACAAATGG
	F-f	TGCGAAGCTGTCGCACAAATGG
**Reverse Primers**	R-a	TGCGCTTGTTGGAACCGTAAC
	R-b	TGCGCTTGTTGGATCCGTAAC
	R-c	TGCGCTTGTTGGAACCATAAC
	R-d	TGCGCTTGTTGGAGCCGTAAC
	R-e	TGCGCTTGTTAGAACCGTAAC
	R-f	TACGCTTGTTGGAACCGTAAC
	R-g	TGCGCTTGTTGGAACCGTAGC
	R-h	TGCGCTTGTTCGATCCGTAAC
	R-i	TGCGCTTGTTGGAGCCATAAC
	R-j	TGCGCTTGTTTGATCCGTAAC
	R-k	TGCGCTTGTTTGAACCATAAC
	R-l	TACGCTTGTTGGAACCATAAC
	R-m	TACGCTTGTTGGAGCCGTAAC
	R-n	TGCGCTGGTTGGAACCATAAC
	R-o	TGAGCTTGTTCGATCCGTAAC
	R-p	TACGCTTGTTGGAGCCATAAC
	R-q	TGAGCTTGTTTGATCCGTAAC

	R-r	TAAGCTTGTTGGATCCGTAGC
	R-s	TCAGCTTGTTGGATCCATAGC

Supplementary Table: q-PCR RpL32 primer pairs for the fly species from the above sequence table

	Species	*F-primer*	*R-primer*
1	*Zaprionus davidi*	F-a	R-c
2	*Zaprionus taronus*	F-a	R-c
3	*Zaprionus tuberculatus*	F-a	R-c
4	*Drosophila putrida*	F-d	R-q
5	*Drosophila micromelanica*	F-a	R-g
6	*Drosophila euronotus*	F-a	R-g
7	*Drosophila americana*	F-c	R-a
8	*Drosophila virilis*	F-c	R-a
9	*Drosophila montana*	F-c	R-a
10	*Drosophila flavomontana*	F-c	R-a
11	*Drosophila lacicola*	F-c	R-a
12	*Drosophila hydei*	F-a	R-a
13	*Drosophila buzzatii*	F-a	R-e
14	*Drosophila arizonae*	F-a	R-a
15	*Hirtodrosophila duncani*	F-f	R-c
16	*Drosophila tropicalis*	F-b	R-k
17	*Drosophila saltans*	F-a	R-n
18	*Drosophila prosaltans*	F-a	R-n
19	*Drosophila subobscura*	F-a	R-i
20	*Drosophila ananassae*	F-f	R-a
21	*Drosophila simulans*	F-d	R-h
22	*Drosophila mauritiana*	F-d	R-h
23	*Drosophila melanogaster*	F-d	R-h
24	*Drosophila santomea*	F-a	R-n
25	*Scaptodrosophila lativittata*	F-a	R-m
26	*Scaptodrosophila lebanonensis*	F-d	R-h
27	*Scaptodrosophila pattersoni*	F-a	R-m

Supplementary Figure: Phylogeny of the 27 host species as constructed by BEAST program, node labels on the phylogeny are posterior supports and the scale bar is the number of substitutions per site

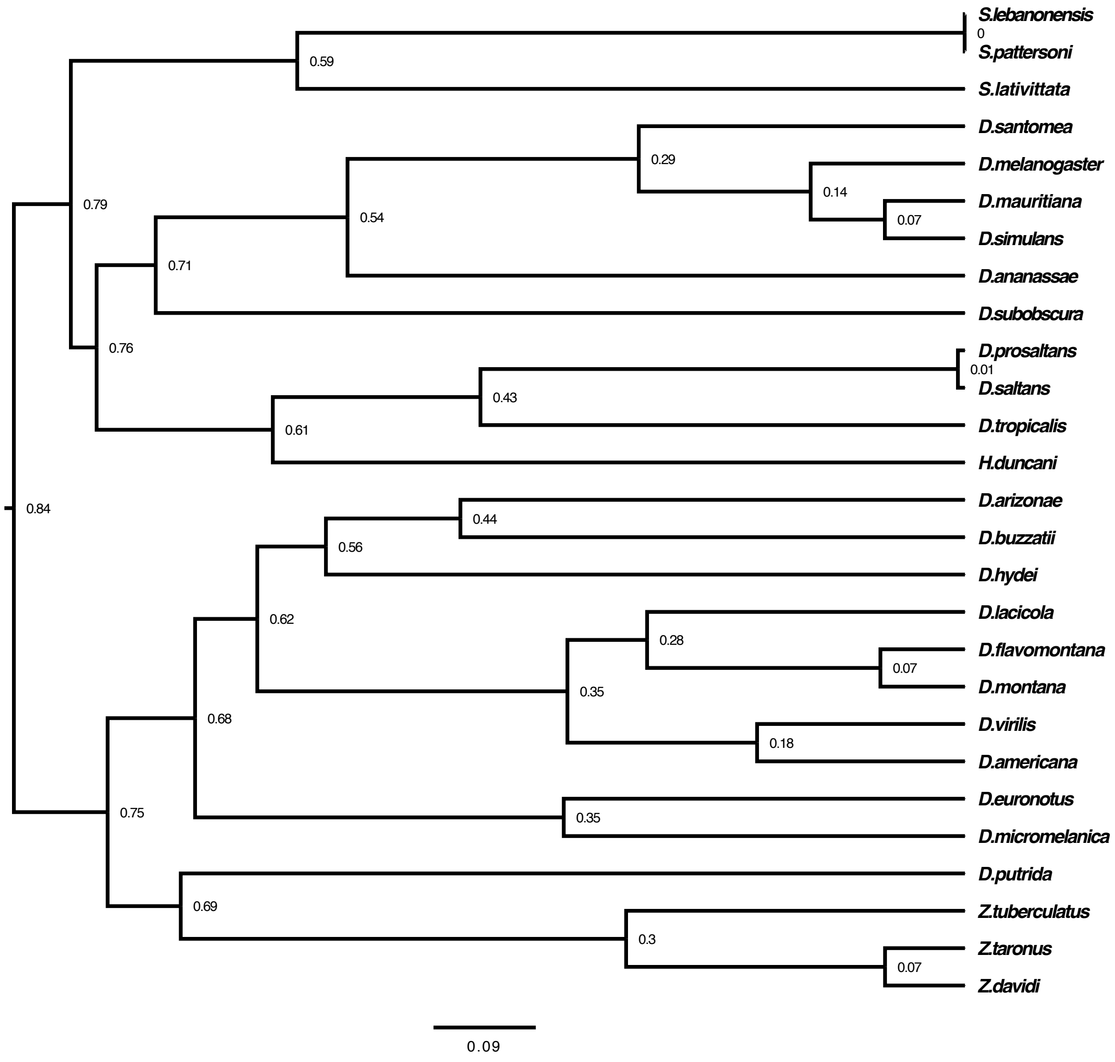

Full project dataset: figshare.com/

- Genbank accession numbers of sequences used to infer the host phylogeny: 10.6084/m9.figshare.13079366
- Full dataset for main analysis: https://doi.org/10.6084/m9.figshare.13079465.v1
- R code: https://doi.org/10.6084/m9.figshare.13079351.v1
- Phylogenetic Tree for analysis: 10.6084/m9.figshare.13079402
