# Supplementary material for: Viral susceptibility across host species is largely independent of dietary protein to carbohydrate ratios
Source: J Evol Biol. 2021 Mar 31;34(5):746–56. doi: 10.1111/jeb.13773 (PMC8436156; doi:10.1111/jeb.13773)
Supplement: Supplementary file 2 — Table S1 [file JEB-34-746-s001.docx]

**TABLE S1.** Ingredients for the experimental diet treatments

| Diet | Ratio Protein: Carb | Cornmeal (g) | Dextrose (g) | Yeast (g) | Agar (g) | Nipagin (ml) | dH20 (L) | Calories per 100ml |
| --- | --- | --- | --- | --- | --- | --- | --- | --- |
| High | **1:5** | **176** | **131.2** | **84** | **22** | **29** | **1** | **142.60** |
| Medium | **1:10** | **176** | **176** | **38** | **22** | **29** | **1** | **142.30** |
| Low | **1:20** | **176** | **203** | **10** | **22** | **29** | **1** | **142.02** |
